# High Quality-Factor and Spectrum-Clean AlN Lamb-Wave Resonators with Optimized Lateral Reflection Boundary Conditions and Transducer Design

**DOI:** 10.3390/mi13050779

**Published:** 2022-05-15

**Authors:** Haiyan Sun, Shitao Lv, Aoyu Zhang, Chenguang Song, Xinyi Sun, Fazeng Tan, Liuhong Liang, Yinfang Zhu, Jicong Zhao

**Affiliations:** 1 Jiangsu Key Laboratory of ASIC Design, Nantong University, Nantong 226019, China; sun.yan@ntu.edu.cn (H.S.); lst@stmail.ntu.edu.cn (S.L.); zay@stmail.ntu.edu.cn (A.Z.); 1811052006@stmail.ntu.edu.cn (X.S.); 2 State Key Laboratories of Transducer Technology, Shanghai 200050, China; 3 School of Electrical Engineering, Nantong University, Nantong 226019, China; songchenguang@ntu.edu.cn; 4 The 26th Research Institute of China Electronics Technology Group Corporation, Chongqing 400060, China; tanfz@cetccq.com.cn (F.T.); lianglh@cetccq.com.cn (L.L.); 5 Institute of Semiconductors, Chinese Academy of Sciences, Beijing 100083, China; yfzhu@semi.ac.cn

**Keywords:** MEMS, Lamb-wave resonator, quality factor, transverse mode, reflection boundary

## Abstract

This paper presents a high quality-factor (*Q*) and spectrum-clean AlN Lamb-wave resonator (LWR). The width of its lateral reflection boundary was optimized to weaken the transverse modes’ coupling and wave guiding, and then to improve the LWR’s *Q* value and spectral purity, which was verified by finite element analysis and experimental characterization. In addition, the series resonance quality factor (*Q_s_*) value of the interdigitated (IDT)-Ground LWR is similar to that of the IDT-Floating LWR, but its parallel resonance quality factor (*Q_p_*) is nearly doubled, due to the reduction of the electrical loss induced by its static capacitance (*C*_0_). The measured results show that the designed LWR with optimized boundary reflection conditions and IDT-Ground structure exhibit *Q_s_* and *Q_p_* values as high as 4019.8 and 839.5 at 401.2 MHz and 402.9 MHz, respectively, meanwhile, it has good spectral purity. Moreover, the influence of the metal ratio and material of the LWR’s IDT electrodes on the device’s performance was also studied by theoretical analysis and experimental verification.

## 1. Introduction

Benefiting from the development of micro-electro-mechanical systems (MEMS) technology and the advanced micro-fabrication process, the MEMS acoustic resonators have been widely used in communication and sensing systems, such as oscillators, filters, and sensors, due to their small size, low power consumption, high performance, and so on [1,2,3]. At present, commercial MEMS acoustic resonators mainly include surface acoustic wave (SAW) resonators, solidly mounted resonators (SMRs) and film bulk acoustic resonators (FBARs) [4,5,6]. SAW resonators have a simple and low-cost manufacturing process and can realize multi-band integration on a single chip. However, SAW resonators can hardly achieve high quality-factor (*Q*) value and large power capacity, and their frequencies are difficult to exceed 3 GHz due to the low phase velocity and lithography limit [7]. SMRs and FBARs have the advantages of high frequency, high *Q* value, and large power capacity, but it is impossible to achieve multi-band on a single-chip due to the thickness of the piezoelectric film determining their resonant frequencies [8]. Recently, AlN Lamb-wave resonators (LWRs) have attracted more and more attention, taking the advantages of both SAW resonators, SMRs, and FBARs: the lithographically defined frequency, high performance, multi-band integration, complementary-metal-oxide-semiconductor (CMOS) compatible fabrication process, and so on [9,10].

For LWRs, their lowest order symmetric (S0) mode is respected for its high phase velocity and weak velocity dispersion [11]. However, some spurious modes existing in the vicinity of the S0 main mode will inevitably deteriorate the LWRs’ performance [12]. The strong transverse spurious modes across the lateral direction may reduce the accuracy and stability for oscillators and sensors, and induced in-band ripples may seriously affect the performance of filters [13]. Until now, there have been two main methods to suppress the transverse spurious modes. One type of method involves changing the edge shapes or the anchors of the resonators to effectively distribute and scatter the transverse acoustic wave [14,15,16]. Another type of method involves applying electrode apodization or dummy electrodes to suppress the transverse modes [17,18]. However, the change of the transducer length caused by the apodization or dummy electrodes may cause the additional loss and reduction of the electromechanical coupling coefficient (*k*^2^*_eff_*).

Furthermore, high *Q* values are highly required for LWRs to achieve the low insertion-loss filters, low phase-noise oscillators, and high-resolution sensors [19,20]. In the past, the researchers of the piezo-MEMS resonators’ *Q* values mainly focused on the series resonance quality factor (*Q_s_*), instead of the parallel-resonance quality factor (*Q_p_*) [21]. The *Q_s_* is mainly determined by the motional resistance (*R_m_*) and the routing resistance (*R_s_*), which represents the acoustic-wave loss and the electrical loss. The *Q_s_* is usually larger than *Q_p_*, because the *R_s_* affected by metal wiring is much smaller. Instead, *Q_p_* is determined by the *R_m_* and the static resistance (*R*_0_), represents the electric loss and other acoustic loss around the parallel resonant frequency (*f_p_*), which can precisely indicate the level of acoustic loss [9].

In this paper, the generation mechanism of the transverse modes in LWRs was theoretically analyzed, and the effective technique to suppress the transverse mode was also proposed. The influence of the boundary reflection structure on the AlN LWRs’ performance was studied. The displacement-field distributions and the admittance responses under different reflection boundary conditions were simulated by using finite element analysis (FEA), and the reflection boundary structure was optimized to change the acoustic reflection conditions so as to suppress the spurious mode. In addition, the LWRs with different electrode structures were analyzed by using FEA simulation and experimental characterization. The amplitudes of transverse spurious modes in the experimental results are consistent with the theoretical prediction and the FEA simulation. Based on the measured frequency responses, the Modified Butterworth—van Dyke (MBVD) equivalent circuit model was constructed to analyze the influence of the change of static capacitance on *Q_p_* under different electrode structures.

## 2. Design and Micro-Fabrication

**Figure 1:**
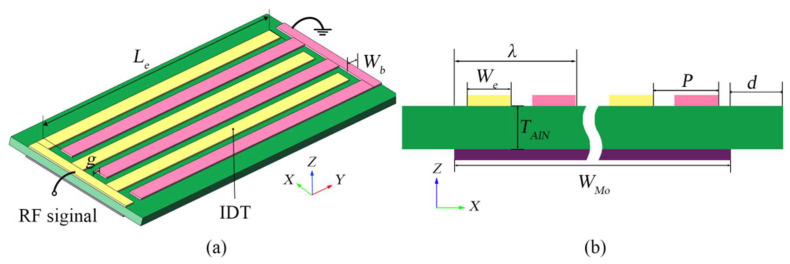
(**a**) Schematic view and (**b**) Cross-section schematic of the designed AlN LWR with extended lateral reflection boundary conditions.

As shown in Figure 1a, the designed AlN Lamb-wave resonator in this work comprises of a 1μm-thick AlN film sandwiched between two patterned metal layers that are used to actuate Lamb-wave modes in the AlN film. The suspended flat edge and bottom cavity are employed as the Lamb-wave reflectors, and the full support structure is used to support the device above the cavity. Figure 1b shows the cross-sectional view of the resonator, and the main geometrical parameters are illustrated in Table 1, mainly including interdigitated (IDT) period (*p*), IDT number (*n*), bottom electrode width (*W_Mo_*), effective electrode length (*L_e_*), bus width (*W_bus_*), and finger-to-bus gap (*g*). The top IDT electrodes are patterned by a 200 nm-thick Al layer, connected to alternate between the ground and RF signals, and the bottom electrodes are patterned by a 200 nm-thick Mo layer. The top IDT electrodes and bottom electrodes are combined to actuate the S0 mode in the AlN film and the frequency of LWRs is mainly dependent on the *p* of IDTs, which can be expressed in Equation (1) [22]. For LWRs, the IDTs’ central regions located at the potential maximums and displacement-amplitude minimums can contribute to achieving ideal harmonic conditions. In this work, three LWRs with different extended lateral reflection boundary widths (*d*) of 3 μm, 6 μm, and 12 μm were designed. The design can avoid the exposure of the Mo electrode at the reflection boundary due to lithography deviations, which may induce the undesired etching of the Mo electrode during XeF_2_ releasing. The suitable extended width can ensure the Lamb-wave reflected in the region of maximum displacement, which can maintain excellent spectral purity and high *Q* value.

(1) $$f=\frac{v}{\lambda }=\frac{v}{2p}$$

where *v* is the phase velocity of the LWR, and λ is the wavelength as shown in Figure 1b.

**Figure 2:**
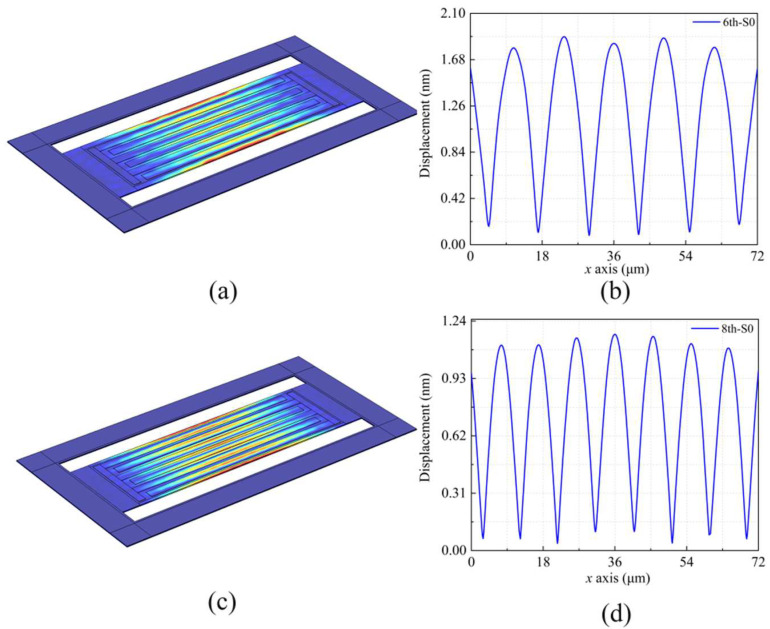
(**a**) Simulated displacement-field distributions, (**b**) displacement curve of the mass point on the *x*-axis for the LWR at 6th-S0 mode, (**c**) simulated displacement-field distributions and (**d**) displacement curve at 8th-S0 mode.

Based on the LWRs’ structural characteristics, the S0 mode can be effectively excited, which has the advantages of high phase velocity, weak phase velocity dispersion, high *Q* value, and medium *k*^2^*_eff_*. However, the S0 mode may coincide with some transverse spurious modes, which will deteriorate the LWRs’ spectral purity. In order to study the transverse spurious modes, the displacement-field distributions of the LWR with the 3-μm-width reflection boundary were derived by using COMSOL Multiphysics V5.5a software. Figure 2a shows the vibration mode at 363.1 MHz, and Figure 2b shows its displacement curve of the mass point along the *x*-axis excluding the extended regions. In Figure 2b, there exist six static-displacement points corresponding to the blue areas under the IDT electrodes. If the vibration mode exists, with the number of static displacement points equaling to *n*, it can be called nth-S0 resonance [23]. Figure 2c,d show the vibration mode and displacement curve at 474.6 MHz, respectively, and the mode can be defined as 8th-S0 resonance. It can be seen from Figure 2b,d that the vibration amplitude of 8th-S0 resonance is smaller than that of 6th-S0 resonance. Therefore, the mode of 8th-S0 resonance is a spurious mode far away from the of S0 main mode. The approximate vibration amplitude *u*(*x*) of nth-S0 mode along the transverse width can be expressed by Equation (2) [24]:

(2) $$u(x)=\frac{\omega_{n}}{2}(cos\frac{2\pi }{W_{AlN}-2d}nx+1)$$

where *ω_n_* is the vibration amplitude of the resonator’s endpoints at *n*th-S0 resonance; *x* is coordinate value along the width direction; *n* is the number of static displacement points.

The reason for the generation of spurious modes (such as the 8th-S0 mode) is that the incident Lamb wave is reflected on the edge reflector, and the amplitude and phase of the reflected wave are deviated from the incident wave, which causes long interference between them. The amplitude and phase deviation are related to the width of the reflection boundary. In other words, choosing an appropriate boundary width can suppress spurious modes near the main mode. The extended lateral boundary reflection widths *d* were set as 3 μm, 6 μm, and 12 μm for different LWRs. By extending lateral reflection boundaries, the acoustic boundaries of the LWRs are modified, and the 6th-S0 and 8th-S0 resonances may be excited together.

**Figure 3:**
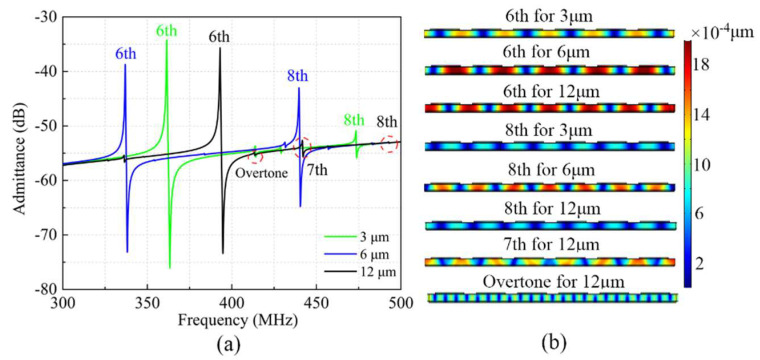
(**a**) The simulated admittance curves and (**b**) displacement diagrams for the LWRs with *d* = 3 μm, 6 μm, and 12 μm.

The COMSOL software was used to calculate the influence of the lateral reflection boundary width on the LWRs’ frequency-response characteristics. As shown in Figure 3a, the 6th-S0 resonant frequency of the LWRs with *d* = 3 μm, 6 μm and 12 μm are 363.1 MHz, 337.9 MHz, and 394.8 MHz, respectively. For the LWR with *d* = 6 μm, the admittance peak-to-peak value of the 8th-S0 resonance reaches up to 33.6 dB, which deteriorates the *Q* value and spectral purity of the 6th-S0 main mode. Fortunately, the 8th-S0 mode can be effectively converted into other Lamb waves by adjusting the width of the lateral reflection boundary. Especially for the LWR with *d* = 12 μm, the 8th-S0 resonance almost disappeared. In order to further analyze the phenomenon, the displacement-field distributions were also extracted in Figure 3b. In the case of *d* = 6 μm, the displacement of the 8th transverse spurious mode is relatively large, and the strong coupling of the spurious mode has caused serious damage to the 6th main mode. As the boundary extension is 3 μm and 12 μm, the displacement amplitude of the 8th spurious mode is significantly reduced. Moreover, the static displacement points of the LWR with *d* = 12 μm are evenly distributed in the middle of each IDT, which can minimize the interface loss caused by the electrode and the loss caused by the acoustic impedance matching between materials. For the LWR with *d* = 12 μm, the 8th-S0 mode is transformed into the 7th-S0 mode and overtone mode affected by the longitudinal mode, and the 8th-S0 mode almost disappears completely. The *k*^2^*_eff_* of the LWRs with *d* = 3 μm, 6 μm and 12 μm are 1.2%, 0.8% and 1.1%, respectively. The slight difference is because the change of the width of the reflection boundary will also have a certain influence on the amplitude and phase of the main mode, and then cause the change of *k*^2^*_eff_*. Moreover, the spurious signal causes a certain attenuation to the *k*^2^*_eff_* of the main mode. The spurious mode of the LWR with *d* = 6 μm is the most obvious, so the *k*^2^*_eff_* is the lowest. Compared with the LWR of *d* = 6 μm, although the LWR of *d* = 12 μm can effectively suppress the 8th-S0 mode, the 7th-S0 mode and overtone mode converted from the 8th-S0 mode also slightly affect the *k*^2^*_eff_* of the main mode.

**Figure 4:**
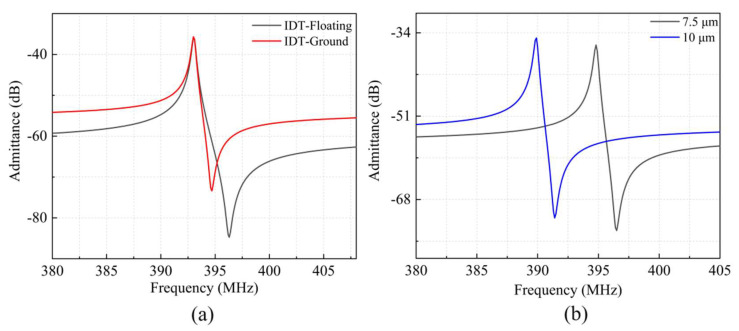
The simulated admittance curves of (**a**) IDT-Floating and IDT-Ground electrode structure, (**b**) 7.5 μm and 10 μm electrode width.

In order to explore the impact of the IDT structure on device performance, the LWRs with IDT-floating and IDT-Ground structures were analyzed in the case of *d* = 12 μm. As shown in Figure 4a, the IDT-Floating electrode structure offers a larger *k*^2^*_eff_* than IDT-Ground because the floating potential offers smaller static capacitance. The electric loss is reduced due to the reduction in static resistance, which means that the IDT-Ground LWR exhibit a higher *Q_p_* value. And the *Q_p_* value is a good indicator of the actual acoustic loss level. Besides, the LWRs with IDT widths *W_e_* of 7.5 μm and 10 μm were also designed to study the effect of *W_e_* on the LWRs’ performance, and the simulated admittance curves are shown in Figure 4b.

Affected by the mass-loading effect, the resonant frequency of the LWR decreases with the increase of *W_e_*. Compared to the above-mentioned LWR with *W_e_* = 8.5 μm, the LWR with *W_e_* = 7.5 μm exhibit higher resonant frequency, and the resonant frequency of the LWR with *W_e_* = 10 μm are decreased. In addition, their *Q* values are both attenuated to a certain extent. A model of the loss mechanism is used to investigate the causes affecting the *Q* decay of the resonator, as shown in Equation (3) [25]:

(3) $$\frac{1}{Q}=\frac{1}{Q_{\mathrm{electric}}}+\frac{1}{Q_{\mathrm{acoustic}}}+\frac{1}{Q_{\mathrm{TED}}}+\frac{1}{Q_{\mathrm{material}}}+\frac{1}{Q_{\mathrm{interface}}}+\frac{1}{Q_{\mathrm{other}}}$$

including electric loss (*Q_electric_*), acoustic loss (*Q_acoustic_*), thermoplastic damping (TED) loss (*Q_TED_*), intrinsic material limitations (*Q_material_*), interface loss (*Q_interface_*), and other loss such as anchor loss and phonon-phonon interaction loss. With the increases of the electrode width, the interface loss between the piezoelectric and electrode layers increases, resulting in a decrease in the *Q_interface_* value, which in turn causes the *Q* value of the resonator with *W_e_* = 10 μm to be lower than that of the resonator with *W_e_* = 8.5 μm. In the case of *W_e_* = 7.5 μm, its *Q* value is also decreased due to the decrease of *Q_electric_* caused by the increase of electric loss. By contrast, the LWR with *W_e_* = 8.5 μm can obtain better frequency-response characteristic.

**Figure 5:**
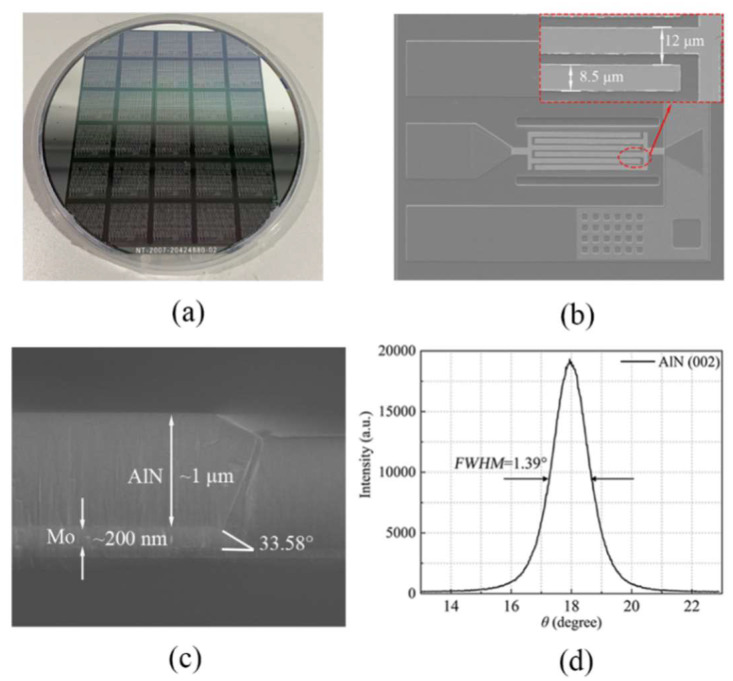
(**a**) The fabricated 6-inch wafer; (**b**) The fabricated AlN LWRs with the extended boundary of 12 μm; (**c**) SEM images of the cross section of the Mo sidewall; (**d**) XRD rocking curve of the 1-μm-thick AlN (002) layer.

The designed LWRs were fabricated on a 6-inch silicon wafer based on seven-step lithography. The fabrication process starts with cavity etching, thermal oxidation, polysilicon deposition, etching and polishing, which forms a release barrier that can prevent the excessive etching during XeF_2_ releasing. The detailed process flow is similar to that described in [26]. The fabricated 6-inch wafer is shown in Figure 5a, and the scanning electron microscope (SEM) image of a fabricated LWR is shown in Figure 5b. Figure 5c shows the cross-sectional view of the composite AlN-seed/Mo/AlN layers. It is clearly seen that the bottom Mo electrodes have a shear angle of 33.58°, which can contribute to a relatively flat surface and no crack for the AlN structural layer. In Figure 5d, the measured X-ray diffraction (XRD) rocking curve of the 1-μm-thick AlN (002) layer has a full width at half maximum (*FWHM*) value of 1.39°, which indicates that the AlN layer has an excellent crystalline quality.

## 3. Measurement and Discussion

**Figure 6:**
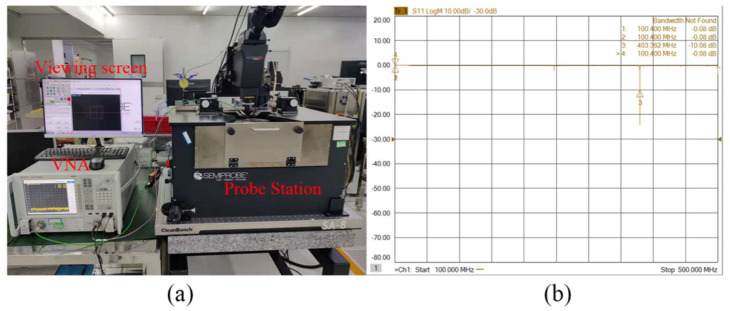
(**a**) Schematic diagram of the testing equipment for the fabricated LWRs; (**b**) measured *S*_11_ spectrum of a fabricated LWR.

The transmission characteristics of the fabricated resonators were measured by a Keysight N5244A vector network analyzer (VNA) and a Cascade SA8 probe station at room temperature and atmospheric pressure, as shown in Figure 6a. The signal power of the VNA was set as 0 dBm (1 mW), and a standard short-load-open-through (SLOT) calibration was performed before testing. The designed devices in this work were all one-port devices, so their frequency responses obtained by the VNA are the reflection scattering parameters *S*_11_, as shown in Figure 6b.

The frequency response of admittance *Y*_11_ can be extracted from the measured *S*_11_ by using Equation (4):

(4) $$Y_{11}\left(\mathrm{dB})=20\mathrm{lg}\right|Y_{11}\left|=20\mathrm{lg}\right|\frac{1}{Z_{0}}\frac{1-S_{11}}{1+S_{11}}|$$

where the *Z*_0_ = 50 Ω represents the characteristic impedance, which is the source or load impedance of the VNA.

**Figure 7:**
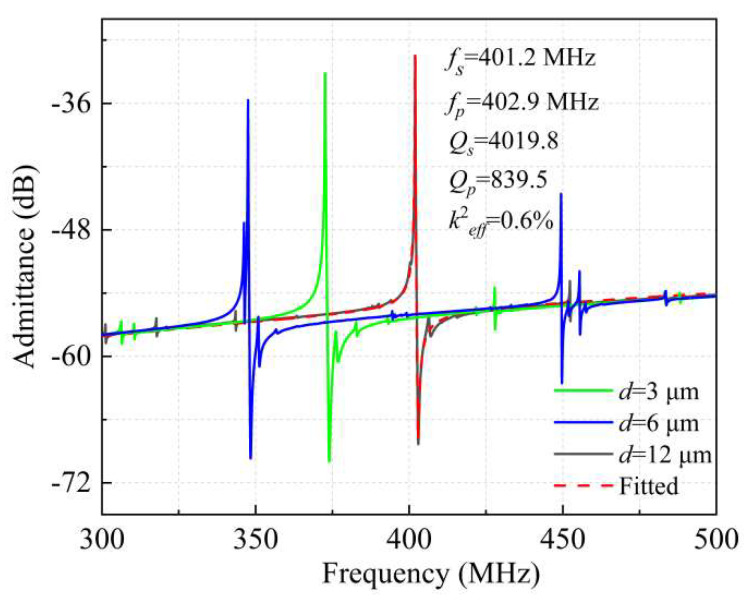
The measured admittance curves for the extended lateral reflection boundary width of 3 μm, 6 μm and 12 μm, and the MBVD fitting curve as *d* = 12 μm.

The admittance curves of the fabricated resonators under different reflection boundary conditions with *d* = 3 μm, 6 μm, and 12 μm were extracted according to the measured *S*_11_, as shown in Figure 7. For the LWRs with *d* = 3 μm and 6 μm, the measured results show that their 6th-S0 resonances occur at 374.8 MHz and 349.5 MHz, respectively, and 8th-S0 resonances occur at 427.7 MHz and 449.3 MHz. The measured resonant frequencies are in good agreement with the calculated values, and the small deviation may be induced by the biases of the geometric dimensions and material parameters between the simulation and fabrication. In accord with the simulation results of Figure 3, in the two LWRs with *d* = 3 μm and 6 μm exist obvious 8th spurious resonances, especially for the LWR with *d* = 6 μm. Due to the good guided waves and limited *L_e_*, the strong coupling of the 8th spurious mode causes an adverse impact on the main mode. By adjusting the reflection boundary width, the transverse propagation path can be changed to weaken the coupling and the wave guiding of the 8th transverse mode. For the LWR with *d* = 12 μm, the spurious mode was effectively converted and dissipated, splitting into multiple longitudinal modes, which is consistent with the simulation results above.

The *Q_s_* values and *k*^2^*_eff_* are important parameters for the LWRs, and both of them were extracted based on the measured results by using Equations (5) and (6) [22]. The *Q_s_* value and the *k*^2^*_eff_* of the LWR with *d* = 12 μm were up to 4019.8 and 0.6%, respectively. For the LWRs with *d* = 3 μm and 6 μm, however, the *Q_s_* values decreased to 3726 and 2870, and the *k*^2^*_eff_* were 0.84% and 0.59% with no significant variation. The *k*^2^*_eff_* of the LWR with *d* = 12 μm was slightly lower than that of the other two LWRs. On the one hand, the widening of the reflection boundary suppresses the spurious modes and also suppresses the main mode to a certain extent. On the other hand, the 8th-S0 mode is transformed into other smaller spurious modes affected by the longitudinal mode, which will cause a decrease in the *k*^2^*_eff_* of the main mode. As *d* = 12 μm, the 8th transverse spurious mode is effectively suppressed due to its ideal harmonic condition, which helps to obtain the pure spectrum. Moreover, the static displacement points are evenly distributed in the middle of the IDTs, which minimizes the interface loss and acoustic loss. Therefore, the designed LWR with *d* = 12 μm can achieve the highest *Q* values, which is consistent well with the above simulation results.

(5) $$Q_{s}=\frac{f_{s}}{\Delta f_{3dB}}$$

(6) $$k_{eff}^{2}=\frac{\pi^{2}}{4}\frac{\left(f_{p}-f_{s}\right)}{f_{p}}$$

**Figure 8:**
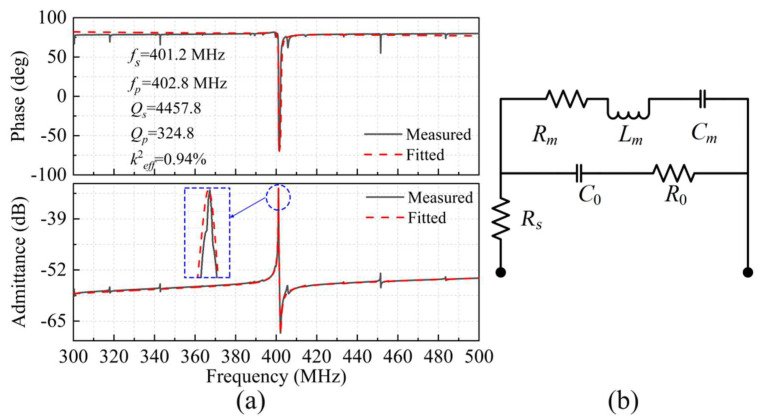
(**a**) The measured and fitted admittance curves and phase curves of the LWR with the IDT-Floating electrode structure, (**b**) the MBVD model used to extract the LWR’s equivalent electrical parameters.

The frequency response of the IDT-Floating resonator with the 12-μm-width reflection boundary was also measured, as shown in Figure 8a. The measured results show its *f_s_* = 401.2 MHz, *f_p_* = 402.8 MHz, and *Q_s_* = 4457.8 are similar as the IDT-Ground resonator with *d* = 12 μm, and its *k*^2^*_eff_* increases from 0.6% to 0.94% due to smaller static-capacitance value. However, its *Q_p_* value significantly drops from 839.5 to 324.8. To further analyze the cause of the *Q_p_* decrease in the IDT-Floating structure, the MBVD model was constructed to fit the measured admittance curves and extract the equivalent circuit parameters, as shown in Figure 8b. The equivalent circuit is composed of a mechanical resonance branch, which includes a motional inductance (*L_m_*), a motional capacitor (*C_m_*), and a motional resistance (*R_m_*) in series, corresponding to the mass, stiffness, and damping of the mechanical system, respectively.

Based on the MBVD model, the extracted equivalent circuit parameters of the LWRs with IDT-Ground and IDT-Floating structures are listed in Table 2. The *R_s_* on the main circuit corresponds to the Ohmic loss of the electrode. *C*_0_ on the other branch is the static capacitance, representing the electrostatic coupling between electrodes, and *R*_0_ represents the electrical losses and other acoustic losses in the piezoelectric film [27]. Compared with the IDT-Floating structure, the LWR with the IDT-Ground structure can provide a larger cross-field capacitance due to its unidirectional vertical electric field, which greatly makes the *C*_0_ larger. The increase of *C*_0_ can effectively reduce the dielectric loss and thus reduce *R*_0_. It can be seen from Equation (7) that the smaller *R*_0_ is the cause of the increase of *Q_p_* in the IDT-Ground structure [28].

(7) $$Q_{p}=\frac{1}{\omega_{p}(R_{m}+R_{0})C_{m}}$$

where *ω_p_* is the angular resonant frequency (*ω_p_* = 2*πf_p_*).

**Figure 9:**
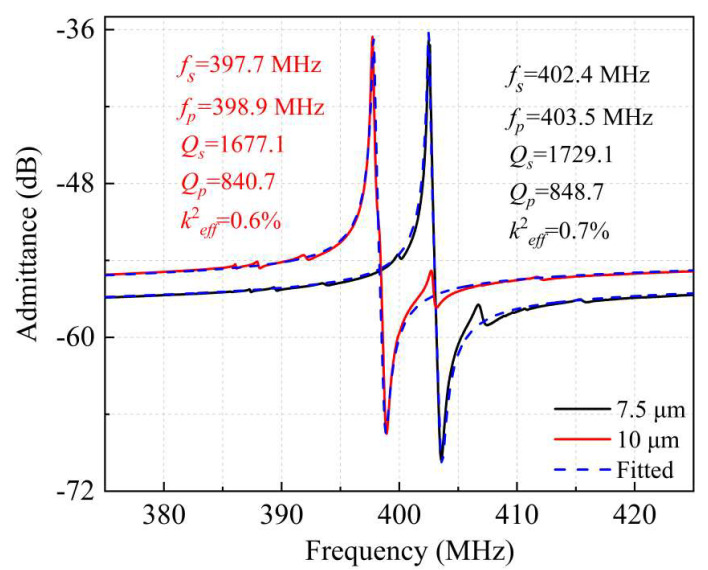
The measured and fitted admittance curves of the resonator with the electrode widths of 7.5 μm and 10 μm.

In addition, under the same boundary reflection conditions, the *W_e_* has a certain impact on the performance of the LWRs. In the case that the reflection boundary was extended for one IDT period with IDT-Ground structure, the LWRs with *W_e_* = 7.5 μm and 10 μm were also measured, as shown in Figure 9. The measured *f_s_* for *W_e_* = 7.5 μm and 10 μm were 402.4 MHz and 397.7 MHz, respectively, which are similar to that of the LWR with *W_e_* = 8.5 μm, and the small deviation is mainly caused by the mass-loading effect. However, the *Q_s_* values of the two LWRs were seriously decreased to 1677.1 and 1729.1, respectively. The relationship between the device’s *Q_s_* value and various losses can be expressed as Equation (3). The extracted equivalent circuit parameters of the LWRs with different *W_e_* are also listed in Table 2. As *W_e_* = 10 μm, the *R_m_* value is increased and then reduces the *Q_s_* value, which is mainly due to the intensification of acoustic loss, intrinsic material loss, and interface loss. For the LWR with *W_e_* = 7.5 μm, its *Q_s_* value is also reduced. With the decrease of *W_e_*, the *C*_0_ value is reduced which leads to a larger dielectric loss, so that the *R_m_* is increased can be seen in Equation (8) [29]. In summary, the LWR with appropriate *W_e_* = 8.5 μm can contribute to achieving the maximum *Q_s_* value, which is consistent with the above simulation results.

(8) $$R_{m}\propto \frac{1}{C_{0}k_{eff}^{2}Q_{s}}$$

**Figure 10:**
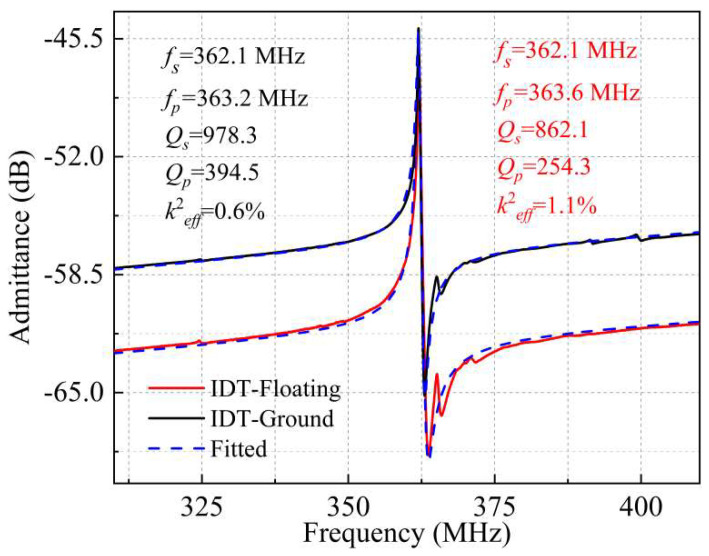
The measured and fitted admittance curves of LWRs using Au as the top electrode material with the extended lateral reflection boundary width of 12 μm.

Furthermore, the LWRs with the Au IDTs have also been fabricated to study the impact of different IDT electrodes materials on the LWRs’ performance. Figure 10 shows the measured and fitted admittance curves of the LWRs with IDT-Ground and IDT-Floating structures, and the parameters of the MBVD fittings for these two cases are listed in Table 2. Compared with the LWRs with Al IDTs, the *f_s_* of LWRs with the Au IDTs reduced from 401.2 MHz to 362.1 MHz, which is that Au can make a larger mass-load effect and a lower phase velocity on the LWRs. Furthermore, the *R_m_* of LWRs with Au IDTs using IDT-Ground and IDT-Floating structures are 198.93 Ω and 210.6 Ω, respectively, which are much higher than that of the LWRs with Al IDTs. On the contrary, their *R_s_* values (1.25 Ω and 2.39 Ω) are lower than that of the LWRs with Al IDTs, due to the better stability and smaller contact resistance of Au IDTs. By comparison, the great increase of *R_m_* values has the dominant influence on their *Q* values, and their *Q* values are 978.3 and 862.1, respectively, which is much smaller than that of the Al IDT LWRs. The acoustic impedance values of AlN, Al, and Au are 1061 C/m^2^, 436 C/m^2^, and 1162 C/m^2^, respectively, and the better acoustic impedance matching between AlN and Au will cause serious acoustic-energy dissipation [30].

The LWRs with the extended lateral reflection boundary width of 12 μm are summarized and compared in Table 3 [31,32,33,34]. With IDT-Ground and IDT-Floating structures, the *f_s_*·*Q_s_* values of the fabricated LWRs can reach 1.61 × 10^12^ and 1.78 × 10^12^, respectively. The Figure of Merit (*FoM =* *Q_s_·k*^2^*_eff_*) is always used to estimate the resonators’ performance. In this work, benefiting from the suppressed 8th-S0 mode by widening the reflection boundary by 12 μm, the *FoM* values of the fabricated resonators are as high as 24.11 and 41.90, respectively.

## 4. Conclusions

In this paper, the LWRs with high quality-factor and spectrum-clean have been reported. The generation mechanism of the transverse modes in LWRs was theoretically analyzed. Through theoretical analysis and experimental verification, it is confirmed that the transverse modes can be effectively suppressed by using the optimal lateral reflection boundary width, which is contributed to by the change of the transverse propagation path to weaken the transverse modes’ coupling and wave guiding. The *Q_s_* value of the IDT-Ground LWR is similar to that of the IDT-Floating LWR, but it has higher *Q_p_* value due to its smaller electric loss, which has been systematically analyzed by using MBVD extracted equivalent electrical parameters. Moreover, the appropriate width of IDT electrodes can minimize the summation of acoustic loss, intrinsic material loss, interface loss and electric loss, conducive to achieving the maximum *Q_s_* value. The LWRs were successfully fabricated by using a process, and the process can define the releasing cavities to prevent the XeF_2_ etching of the inactive region. The measured results show that the optimized LWR can obtain *Q_s_* and *Q_p_* values as high as 4019.8 and 839.5, respectively, meanwhile, it has good spectral purity. Compared to the Au-IDT LWR, the Al-IDT LWR can achieve higher *Q* values due to its smaller acoustic loss, which is mainly contributed to by a higher degree of acoustic impedance mismatch between Al and AlN.

## Figures and Tables

**Table 1:** The main geometric dimensions of the designed AlN LWRs.

**Top IDT period (*p*)**	12 μm	**IDT width (*W_e_*)**	8.5 μm
**IDT numbers (*n*)**	6	**Effective electrode length (*L_e_*)**	192 μm
**Bottom electrode width (*W_Mo_*)**	72 μm	**Finger-to-bus gap (*g*)**	6 μm
**AlN plate width (*W_AlN_*)**	96 μm	**Bus width (*W_bus_*)**	7 μm

**Table 2:** The extracted equivalent circuit parameters for the designed LWRs.

Transducer Configurations	IDTs Material	*W_e_* (μm)	*R*_0_ (Ω)	*C*_0_ (fF)	*R_m_* (Ω)	*C_m_* (fF)	*L_m_* (μH)	*R_s_* (Ω)
IDT-Ground	Al	8.5	120.21	663.15	29.91	3.03	51.66	7.33
IDT-Floating	Al	8.5	311.14	188.08	25.31	3.03	192.41	11.25
IDT-Ground	Al	7.5	105.05	595.51	41.48	3.19	48.95	23.65
IDT-Ground	Al	10	74.05	715.95	64.06	3.55	45.08	5.47
IDT-Floating	Au	8.5	378.0	374.31	210.6	2.77	69.82	2.39
IDT-Ground	Au	8.5	106.04	624.21	198.93	3.11	62.12	1.25

**Table 3:** The key performance parameters of the fabricated resonators, and compared with previous ones.

	Ref. [31]	Ref. [32]	Ref. [33]	Ref. [34]	This Work	This Work
*f_s_* (MHz)	457.1	400.3	483	504	401.2	401.2
*Q_s_*	1150	1482	1750	1647	4019.8	4457.8
*k* ^2^ *_eff_*	1.8%	1.19%	1.0%	1.24%	0.6%	0.94%
*f_s_*·*Q_s_*	5.25 × 10^11^	5.93 × 10^11^	8.45 × 10^11^	8.18 × 10^11^	16.1 × 10^11^	17.9 × 10^11^
*FoM*	20.7	17.6	17.5	20.4	24.1	41.9

## References

[1] Liu C.S., Tabrizian R., Ayazi F. (2018). A +/− 0.3 ppm Oven-Controlled MEMS Oscillator Using Structural Resistance-Based Temperature Sensing. IEEE Trans. Ultrason. Ferroelectr. Freq. Control.

[2] Lannacci J. (2018). Internet of things (IoT); internet of everything (IoE); tactile internet; 5G-A (not so evanescent) unifying vision empowered by EH-MEMS (energy harvesting MEMS) and RF-MEMS (radio frequency MEMS). Sensor. Actuat. A-Phys..

[3] Zhu Y., Wang N., Chua G.L., Sun C.L., Singh N., Gu Y.D. (2017). ScAlN-Based LCAT Mode Resonators Above 2 GHz With High FOM and Reduced Fabrication Complexity. IEEE Electron Device Lett..

[4] Agostini M., Cecchini M. (2021). Ultra-high-frequency (UHF) surface-acoustic-wave (SAW) microfluidics and biosensors. Nanotechnology.

[5] Yim M., Jeon B., Yoon G. (2020). Feasibility Study of Small-Sized FBAR-Based Bandpass Filter Covering Digital Dividend Band for LTE Services. J. Semicond. Technol. Sci..

[6] Ma L., Zhang B., Guo X., Wang P., Liu Q.F., Zheng K., Li X.L., Wang J.B. (2021). Bulk acoustic wave resonance characteristic modified by reducing the defects in ZnO-based solidly mounted resonators. Mat. Sci. Semicon. Proc..

[7] He X.L., Guo H.W., Chen J.K., Wang W.B., Xuan W.P., Xu Y., Luo J.K. (2014). Bendable ZnO thin film surface acoustic wave devices on polyethylene terephthalate substrate. Appl. Phys. Lett..

[8] Yantchev V., Katardjiev I. (2013). Thin film Lamb wave resonators in frequency control and sensing applications: A review. J. Micromech. Microeng..

[9] Zou J., Gao A., Pisano A.P. (2020). Ultralow Acoustic Loss Micromachined Butterfly Lamb Wave Resonators on AlN Plates. IEEE Trans. Ultrason. Ferroelectr. Freq. Control.

[10] Daruwalla A., Wen H.R., Liu C.S., Ayazi F. (2020). Low motional impedance distributed Lame mode resonators for high frequency timing applications. Microsyst. Nanoeng..

[11] Yen T.T., Lin C.M., Lai Y.J., Wittwer D., Hopcroft M.A., Pisano A.P. Fine frequency selection technique for aluminum nitride Lamb wave resonators. Proceedings of the 2010 IEEE International Frequency Control Symposium.

[12] Rinaldi M., Zuniga C., Zuo C.J., Piazza G. (2010). Super-high-frequency two-port AlN contour-mode resonators for RF applications. IEEE Trans. Ultrason. Ferroelectr. Freq. Control.

[13] Kim H.J., Segovia-Fernandez J., Piazza G. (2017). The Impact of Damping on Flicker Frequency Noise of AlN Piezoelectric MEMS Resonators. J. Microelectromech. Syst..

[14] Song Y.H., Gong S. (2015). Elimination of Spurious Modes in SH0 Lithium Niobate Laterally Vibrating Resonators. IEEE Electron Device Lett..

[15] Colombo L., Kochhar A., Vidal-Alvarez G., Piazza G. (2018). X-Cut Lithium Niobate Laterally Vibrating MEMS Resonator With Figure of Merit of 1560. J. Microelectromech. Syst..

[16] Pop F.V., Kochhar A.S., Vidal-Alvarez G., Piazza G. (2018). Investigation of Electromechanical Coupling and Quality Factor of X-Cut Lithium Niobate Laterally Vibrating Resonators Operating Around 400 MHz. J. Microelectromech. Syst..

[17] Zhu Y., Wang N., Chua G.L., Chen B.T., Srinivas M., Singh N., Gu Y.D. Apodization Technique for Effective Spurious Mode Suppression of Aluminum Nitride Lamb Wave Resonators. Proceedings of the 2018 IEEE International Ultrasonics Symposium (IUS).

[18] Chen G.F., Casella C., Wu T., Rinaldi M. Single-Chip Multi-Frequency Wideband Filters Based on Aluminum Nitride Cross-Sectional Lame Mode Resonators With Thick and Apodized Electrodes. Proceedings of the 31st IEEE International Conference on Micro Electro Mechanical Systems, MEMS 2018.

[19] Lin C.M., Zou J., Chen Y.Y., Pisano A.P. High-Q Piezoelectric Lamb Wave Resonators Based on AlN Plates with Chamfered Corners. Proceedings of the Ultrasonics Symposium IEEE.

[20] Zhu Y., Wang N., Sun C.L., Merugu S., Singh N., Gu Y.D. (2016). A High Coupling Coefficient 2.3-GHz AlN Resonator for High Band LTE Filtering Application. IEEE Electron Device Lett..

[21] Wang Y., Goh W.L., Chai K.T.C., Mu X.J., Hong Y., Kropelnicki P., Je M. (2016). Parasitic analysis and pi-type Butterworth-Van Dyke model for complementary-metal-oxide-semiconductor Lamb wave resonator with accurate two-port Y-parameter characterizations. Rev. Sci. Instrum..

[22] Liu J.Y., Zhou J., Zhou Y., Gao C., Xie Y., Sun C.L. AlN Checker-Mode Resonators with Routing Structures. Proceedings of the 2019 IEEE International Ultrasonics Symposium.

[23] Gao A., Zou J. Extremely High Q AlN Lamb Wave Resonators Implemented by Weighted Electrodes. Proceedings of the 2019 IEEE International Electron Devices Meeting.

[24] Kaajakari V., Mattila T., Lipsanen A., Oja A. (2004). Nonlinear mechanical effects in silicon longitudinal mode beam resonators. Sensor. Actuat. A-Phys..

[25] Segovia-Fernandez J., Cremonesi M., Cassella C., Frangi A., Piazza G. (2015). Anchor losses in AlN contour mode resonators. J. Microelectromech. Syst..

[26] Zhao J.C., Zhu Z., Sun H.Y., Lv S.T., Wang X.Y., Song C.G. (2021). A MEMS Fabrication Process with Thermal-Oxide Releasing Barriers and Polysilicon Sacrificial Layers for AlN Lamb-Wave Resonators to Achieve f_s_∙Q_m_ > 3.42 × 10^12^. Micromachines.

[27] Zuo C.J., Sinha N., Van der Spiegel J., Piazza G. (2010). Multifrequency Pierce Oscillators Based on Piezoelectric AlN Contour-Mode MEMS Technology. J. Microelectromech. Syst..

[28] Lin C.M., Yantchev V., Zou J., Chen Y.Y., Pisano A.P. (2014). Micromachined One-Port Aluminum Nitride Lamb Wave Resonators Utilizing the Lowest-Order Symmetric Mode. J. Microelectromech. Syst..

[29] Rinaldi M., Hui Y., Zuniga C., Tazzoli A., Piazza G. High Frequency AlN MEMS Resonators with Integrated Nano Hot Plate for Temperature Controlled Operation. Proceedings of the IEEE Frequency Control Symposium.

[30] Bjurstrom J., Wingqvist G., Yantchev V., Katardjiev I. (2007). Temperature compensation of liquid FBAR sensors. J. Micromech. Microeng..

[31] Bassirian P., Moody J., Lu R.C., Gao A.M., Manzaneque T., Roy A., Barker N., Calhoun B., Gong S.B., Bowers S.M. (2019). Nanowatt-Level Wake up Receiver Front Ends Using MEMS Resonators for Impedance Transformation. IEEE Trans. Microw. Theory.

[32] Luo Z., Shao S., Wu T. (2021). Al_0.78_Sc_0.22_N Lamb Wave Contour Mode Resonators. IEEE Trans. Ultrason. Ferroelectr. Freq. Control.

[33] Rinaldi M., Zuo C.J., Spiegel J., Piazza G. (2011). Reconfigurable CMOS Oscillator Based on Multifrequency AlN Contour-Mode MEMS Resonators. IEEE Trans. Electron Dev..

[34] Lu R.C., Manzaneque T., Breen M., Gao A.M., Gong S.B. Piezoelectric RF resonant voltage amplifiers for IoT applications. Proceedings of the 2016 IEEE MTT-S International Microwave Symposium (IMS).

